# The effects of disturbance threat on leaf-cutting ant colonies: a laboratory study

**DOI:** 10.1007/s00040-016-0513-z

**Published:** 2016-09-15

**Authors:** V. C. Norman, T. Pamminger, W. O. H. Hughes

**Affiliations:** School of Life Sciences, University of Sussex, Brighton, East Sussex BN1 9QG UK

**Keywords:** Phenotypic plasticity, Animal behavior, Castes, Social insect, Division of labor, Animal personality, Behavioral syndromes

## Abstract

The flexibility of organisms to respond plastically to their environment is fundamental to their fitness and evolutionary success. Social insects provide some of the most impressive examples of plasticity, with individuals exhibiting behavioral and sometimes morphological adaptations for their specific roles in the colony, such as large soldiers for nest defense. However, with the exception of the honey bee model organism, there has been little investigation of the nature and effects of environmental stimuli thought to instigate alternative phenotypes in social insects. Here, we investigate the effect of repeated threat disturbance over a prolonged (17 month) period on both behavioral and morphological phenotypes, using phenotypically plastic leaf-cutting ants (*Atta colombica*) as a model system. We found a rapid impact of threat disturbance on the behavioral phenotype of individuals within threat-disturbed colonies becoming more aggressive, threat responsive, and phototactic within as little as 2 weeks. We found no effect of threat disturbance on morphological phenotypes, potentially, because constraints such as resource limitation outweighed the benefit for colonies of producing larger individuals. The results suggest that plasticity in behavioral phenotypes can enable insect societies to respond to threats even when constraints prevent alteration of morphological phenotypes.

**Electronic supplementary material:**

The online version of this article (doi:10.1007/s00040-016-0513-z) contains supplementary material, which is available to authorized users.

## Introduction

The ability of organisms to respond flexibly to their environment is fundamental to their evolutionary success. Adaptation towards a locally optimal phenotype can increase both direct and indirect fitness (Via and Lande 1985; Lande 1985). One way in which this can occur is via the production of size variation in response to environmental conditions, such as climate or competition, which is seen in a wide variety of organisms found over an environmental gradient (Rosenzweig 1968; McNab 1971; Lomolino 2005). However, many organisms show other morphological and behavioral adaptations to environmental pressures that enable individuals to increase their fitness over the course of their lifetime (Boag and Grant 1981; Engel and Tollrian 2009; Torres-Dowdall et al. 2012). Understanding the biotic or abiotic environmental stimuli involved in regulating the modification of the morphological or behavioral phenotype, and any potential negative implications associated with this, is central to our understanding of the evolution and dynamics of phenotypic plasticity.

Some of the most extreme examples of phenotypic plasticity are provided by the social insects. In these societies it is often thought that the specialization of individuals into behavioral and sometimes morphological phenotypes (castes) may make them better adapted to their particular roles in the colony, thereby enhancing the division of labor that is commonly thought to be a key to their evolutionary success (Oster and Wilson 1978; Bourke and Franks 1995). However, the degree to which specialists actually outperform generalists is still highly contentious and our understanding of these processes remains limited (Dornhaus 2008; Wright et al. 2014; Gordon 2015). Individual workers are more-or-less sterile in species with advanced societies, such as *Atta* leaf-cutting ants, and therefore gain their fitness indirectly, meaning that selection may act simultaneously at both the individual and colony level, such that individual-level and colony-level optimal caste ratios can be hypothesized to be the same (Hamilton 1964; West-Eberhard 1989; Korb and Heinze 2004). The morphological phenotype of adult social insects is determined during development in the eusocial Hymenoptera (ants, some bees, and some wasps). Both morphological and indeed behavioral phenotypes are not simply determined by environmental conditions such as nutrition and temperature (Wheeler 1986; Kamakura 2011), or by genotype (Robinson and Page 1988; Hughes et al. 2003; Smith et al. 2008; Waddington et al. 2010), but rather by the interaction of genotype and environment (Chapman et al. 2007; Hughes and Boomsma 2007; Schwander et al. 2010). Surprisingly, however, we still have only a limited understanding of how environmental conditions can drive the specialization of different individuals. It is well established that social insect colonies vary in their frequency distributions of morphological phenotypes or behavioral phenotype profiles (Yang et al. 2004; Wray et al. 2011; Chapman et al. 2011; Scharf et al. 2012; Pinter-Wollman et al. 2012; Gordon et al. 2013; Jandt et al. 2014; Wiernasz et al. 2014; Wills et al. 2014). However, our understanding of the environmental conditions generating such intercolony variation is still limited (Hui and Pinter-Wollman 2014; Keiser et al. 2014; Jandt et al. 2014).

Honey bees appear to show differing behavioral phenotypes corresponding to length of disturbance, with short-term disturbance causing an upregulation in aggression (Couvillon et al. 2008), but long-term disturbance decreasing aggressive responses (Rittschof and Robinson 2013). Environmental factors, such as competition, or indeed predation, are also thought to be important in morphological phenotype production in other eusocial insects such as polyembryonic wasp, termite, and social aphid species which show a morphologically specialized soldier caste (Crespi 1992; Shibao 1998; Harvey et al. 2000; Shingleton and Foster 2000; Thorne et al. 2003; Smith et al. 2010). In one of the only direct experimental investigations in ants, Passera et al. (1996) demonstrated that exposure to volatiles from non-nestmate potential competitors caused colonies of *Pheidole pallidula* ants to produce more soldiers within a remarkably short 7-week period. However, the environmental stimuli that interact with genotype to produce different morphological or behavioral phenotypes in other social insects are unknown.

Here, we test experimentally the effect of a controlled environmental stimulus of threat disturbance on morphological and behavioral phenotypes in phenotypically plastic *Atta* leaf-cutting ant societies. *Atta* is one of the most polymorphic of all ant species, with larger workers including specialized soldiers playing the primary role in defending their colonies against threats (Wilson 1980; Whitehouse and Jaffe 1996; Hernández et al. 2002; Hölldobler and Wilson 2010). We test whether repeated threat disturbance leads to individuals within colonies adjusting their behavioral phenotype, the production of morphological phenotypes, or both. We also explore if other traits are affected by such threat disturbance.

## Materials and methods

Immature *Atta colombica* colonies, approximately 12-months-old, were collected from Gamboa, Panama in May 2013. At this age, colonies are too small to produce soldiers (Weber 1972). All colonies were kept in a controlled environment room at the University of Sussex at 80 ± 5 % relative humidity, 26 ± 2 °C and a 12:12 h light/dark cycle, and fed twice weekly on privet leaves (*Ligustrum* spp.) placed in a foraging pot (100 mm × 80 mm × 60 mm), with water provided ad libitum. The mutualistic fungal gardens were housed in plastic boxes (115 mm × 75 mm × 75 mm) covered with a flower pot to maintain a dark and humid environment. The 13 colonies were randomly assigned to either a threat disturbance (6 colonies) or control treatment (7 colonies) group. The colonies were of similar size at the start of the experiment (mean ± s.e. volume of fungus garden: threat-disturbed colonies 107 ± 23.9 ml and control colonies 156 ± 39 ml) and did not differ significantly in size throughout the experiment (Fig. S3). At the start of the experiment, no soldiers or large workers (>2 mm head width) were observed in any of the colonies. Colonies undergoing the threat disturbance treatment had their fungal gardens exposed by removing the flower pot and plastic box lid (85 mm depth) for 2 min. Preliminary trials suggested that this exposure protocol produced a maximal alarm response (increased activity and mandible gaping from workers indicating a response to a threat) from the ants within the 2-min-period. The disturbance was carried out on 4–5 days per week for 17 months, while control colonies were not disturbed in this way. Exposure of the vulnerable fungal garden and brood in this way would only occur in nature during a vertebrate predation attempt (such as by armadillos) and, regardless of cause, would represent a serious threat to colony survival, stimulating a dramatic defensive response in leaf-cutting ant colonies in nature (Wilson 1980; Whitehouse and Jaffe 1996; Rao 2000). The long 17-month-period gave colonies ample time to alter the production of morphological phenotopes. Furthermore, given the development time in *Atta* is about 2 months (Weber 1972) and that Passera et al. (1996) found changes in caste ratios after only 7 weeks, any changes in morphological phenotype should be present after this time. At the end of the disturbance period, the morphological and behavioral phenotypes of colonies were determined. After the end of this long-term experiment, we also carried out a shorter disturbance experiment to test at a finer-scale how quickly colonies could upregulate and downregulate their behavioral responses.

## Alteration of morphological phenotype

To determine if threat disturbance resulted in colonies producing larger individuals, the 50 largest workers from each colony after the 17 month disturbance period were photographed dorsally using a Canon EOS 350d dSLR camera and Canon EF 100 mm f/2.8 Macro lens under constant lighting conditions. Images were imported into Image J and the size of each individual was quantified by measuring the width of the head between the eyes, a commonly used measurement of size in ants, including *Atta* (Wilson 1980; Hölldobler and Wilson 1990; Hughes and Goulson 2001; Holman et al. 2011). The colonies were too young and small to produce soldiers in significant numbers, but we also counted any soldiers, or large workers (2–3 mm head width), present to compare the numbers of soldiers between treatment and control colonies.

## Alteration of behavioral phenotype

During the last month of the long-term disturbance, assays were carried out to compare behavioral phenotypes of individuals within colonies that had either been disturbed or not. To see if threat disturbance affected the responsiveness of ants to threats, we carried out a mandible opening response (MOR) assay (Guerrieri and D’Ettorre 2008; Norman et al. 2014). Ants were chilled on ice until immobile and then harnessed in 0.2 ml pipette tips (Starlab, Bucks, UK), cut at the apex through which the ant’s head was passed and secured with a thin strip of masking tape. Ants were left for 2 h in the harness to acclimatize before being assayed. Three threat treatments were tested in a random order on each ant: a freshly freeze-killed nestmate, a freshly killed non-nestmate (*Acromyrmex echinatior*) or a burst of carbon dioxide. The latter treatment has been used previously as specific stimulus for sampling defensive workers in *Atta* colonies by simulating a vertebrate predation threat (Wilson 1980; Hölldobler and Wilson 2010). The stimulus ants or carbon dioxide burst were gently placed in contact with an antenna of the focal ant for 10 s, and the response of the focal ant recorded as either opening its mandibles for >1 s (a positive MOR), or not responding (Norman et al. 2014). For focal ants that showed a positive MOR, the duration of the response was also recorded. For each colony, this assay was carried out for randomly selected soldiers (>3 mm head width), large workers (2–3 mm head width), medium-sized workers (1.2–2.0 mm head width), and small workers (<1.2 mm head width) to test if specific castes responded differently to defensive stimuli. Five individuals of each caste were tested in each colony, or as many as the colony had for colonies which had very few large workers.

To determine the phototaxis of ants, workers were placed individually in a 90 mm Petri dish, half of which had been covered with black tape (Norman and Hughes 2016). The ants were allowed to acclimatize for 5 min and then filmed for the subsequent 10 min. The proportion of time spent in the light half of the Petri dish was recorded for each individual. This was repeated for 120 ants from threat-disturbed colonies and 140 ants from control colonies (20 per colony in both cases), using randomly selected medium-sized and medium-aged external workers. Worker age correlates positively with a darkening of the cuticular coloration (Armitage and Boomsma 2010), therefore medium-aged ants could be distinguished by their coloration. To see if threat disturbance affected the aggressiveness of ants, individual ants were carefully touched on the head with the tip of a toothpick, similar to Pamminger et al. (2014). The reaction of the ant was ranked (0 = ignore, 1 = antennate, 2 = gape mandibles in a threat response, 3 = bite). This was repeated for 120 ants from the disturbed colonies and 140 ants from the control colonies (20 ants per colony in both cases), using randomly selected medium-sized and medium-aged external workers to control for any differences between castes in aggression. Assays were carried out in the order listed above during the final month of the 17 month disturbance. Ants were returned to the colony after the assays with at least 5 days occurring between assays. Given the number of workers per colony [ca. 5000, of which ca. 3000 would be medias, for colonies of the size used here (Weber 1972)], and that at least 5 days was left between assays, the likelihood of resampling the same ant for multiple assays was very low.

## Potential effects on other traits

To explore whether changes in the behavioral or morphological phenotypes of individuals in response to disturbance might affect other traits in ways that could be potentially negative, we compared the foraging rate, individual immunity, and brood care propensity of ants from threat-disturbed and control colonies. With the exception of the immunity assays, ants were returned to their colonies after use; given the number of workers per colony and that at least 5 days occurred between assays, it was unlikely that ants were resampled for multiple assays.

Foraging rate was quantified 4 days following the last feed. The foraging pot of each colony was filled with leaves and the initial mass of the pot recorded. Each pot was then placed back with its respective colony for 1 h, after which the ants within the pots were removed and counted, and the pots reweighed to determine the proportion of leaf material that had been foraged during the 1 h foraging period. This was carried out once for each colony.

To quantify brood care propensity, individual ants were placed in a 90 mm Petri dish with a randomly selected nestmate larva, allowed to acclimatize for 5 min and then filmed for 10 min. The proportion of time spent interacting with larvae during the 10 min period was recorded. This was repeated with 20 medium-sized and medium-aged external workers from each colony. Using external workers avoided the disruption to the fungus chamber that sampling within-nest workers would have involved and external workers have been shown previously in many ant species to pick up brood found outside of the nest and to transport it back into the colony (Robinson et al. 2012; Tragust et al. 2013).

To determine the effect on individual-level immunity, we measured levels of the phenoloxidase (PO) and prophenoloxidase (PPO) immune enzymes in haemolymph. Haemolymph samples of 1 µl were collected from individual, freeze-killed ants using a calibrated, pulled glass capillary inserted under the cuticle of the thorax. Haemolymph was diluted 1:40 in ice-cold sodium cacodilate/CaCl_2_ buffer (0.01 M Na-Cac, 0.005 M CaCl_2_), flash frozen in liquid nitrogen and stored at −80 °C to disrupt haemocyte membranes and release cellular PPO. All reactions were prepared in 96-well plates on ice. 15 μl of diluted sample was placed in an individual well, together with 5 μl of distilled water for PO reactions or 5 μl of the activation agent alpha-chymotrypsin (5 mg ml^−1^, in distilled water; Sigma Aldrich™) for PPO reactions. Samples were then incubated for 5 min at room temperature. To start the reaction, 35 μl of L-DOPA (4 mg ml^−1^ in distilled water; prepared freshly and protected from light, Sigma Aldrich™) was added to each well and the plate was placed in a Molecular Devices VersaMax micro-plate reader. Temperature was set to 30 °C and the absorbance of each sample at 492 nm was measured every 15 s over a period of 45 min using SoftMax Pro software. For each sample, the enzyme activity was calculated at the maximum slope (Vmax) in the linear phase of the reaction (usually 200–1000 s after the start of the reaction). Each plate had a control well, which contained only buffer and no sample, and all controls displayed essentially no enzyme activity during the reaction (<0.1 mOD min^−1^). Two technical replicates were carried out of each reaction and all samples where the reaction curved showed irregularities were excluded, leaving measurements of PO and PPO for 58 and 67 ants from disturbed colonies, and 50 and 63 ants from control colonies, respectively.

## Speed of behavioral plasticity

To see how quickly *Atta colombica* colonies altered their behavioral phenotype to disturbance, we carried out a finer-scale threat disturbance experiment. Four months after the end of the long-term experiment (the length of at least one generation of adult workers (Weber 1972), the remaining seven control colonies were split into equally sized subcolonies (ca. 500 ml fungus per subcolony) that were randomly assigned to either short-term disturbance or control group. Colonies were monogynous so the queen-right subcolony was randomly assigned between treatments. Disturbed subcolonies were disturbed in the same way as in the long-term experiment but for a period of only 2 weeks, with the behavioral phenotype of colonies being determined using the phototactic (*N* = 126 per treatment group; 18 ants per subcolony), aggression (*N* = 140 per treatment group; 20 ants per subcolony), and MOR assays using two stimuli treatment of a nestmate and a non-nestmate (*N* = 126 and *N* = 136 for the threat-disturbed and control treatment groups, respectively; 18–20 ants per subcolony). Colonies were then left undisturbed for a period of 2 weeks after which the assays were repeated with the same numbers of individuals to determine if colonies would then downregulate behaviors to match their prevailing environmental conditions.

### Statistical analyses

The size and behaviors of ants were compared between threat-disturbed and control colonies using generalized linear mixed models (GLMM), which included colony-of-origin as a random factor. The head width of ants, length of MOR, phototaxis, brood care, and proportion of leaves harvested in the foraging assays were compared using models with gamma distributions and log link function, while the propensities of ants to exhibit a MOR or aggressive response were compared with a binomial distribution and logit link function, and aggression scores using a multinomial distribution and probit link function. The number of ants in each foraging pot in the foraging assay was analyzed using a Poisson distribution with a log link function. Levels of PO and PPO were both log transformed to ensure normality and analyzed in a GLMM with a Gaussian distribution and identity link function. Colony size, measured as volume of fungus garden, was included as a covariate in all models to control for variation in colony sizes. Best fitting models were selected by comparison of AIC values. Overdispersion was checked for in all cases by calculating a dispersion parameter and none of the models was overdispersed. Nonsignificant interaction terms were removed stepwise in all cases to obtain the minimum adequate models. All statistics were performed in SPSS (v.20 SPSS Inc., Chicago, IL, USA).

## Results

### Long-term disturbance

**Fig. 1:**
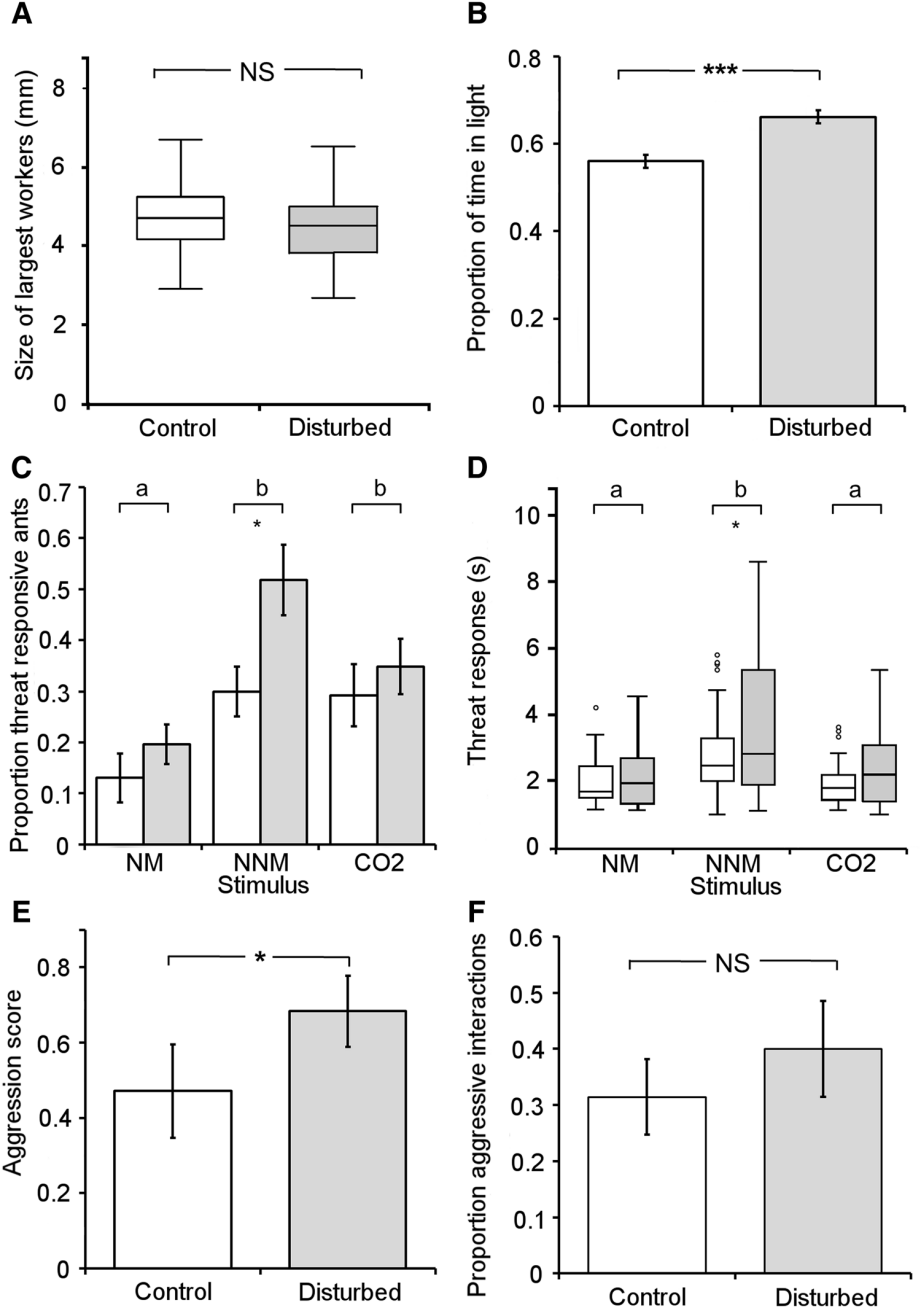
The mean ± s.e. morphological and behavioral phenotypes of *Atta* leaf-cutting ant workers from threat-disturbed colonies (*gray*) and control colonies (*white*): **a** head width of the 50 largest workers in each colony, **b** proportion of time spent in the light half of a half-blackened Petri dish, **c** proportion of positive mandible opening responses (MORs) to threat stimuli, **d** length of MOR to three threat stimuli (*different letters* indicate significantly different responses between treatment stimuli), **e** aggression score (ranging from 0 = no aggression to 3 = bite), and **f** proportion of aggressive interactions. Colonies either received a substantial threat-disturbance every week for 17 months or were not disturbed in this way (control colonies). *Asterisks* indicate a significant difference between threat-disturbed and control colonies (*P < 0.05, **P < 0.01, ***P < 0.001)

There was no significant difference in the size of the largest 50 workers in the threat-disturbed and control colonies (*F*_1,648_ = 0.665; *P* = 0.415; Fig. 1a). The number of soldiers produced was minimal over the 17 month experimental period (4 and 8 from all threat-disturbed and control colonies respectively). There were no differences between treatment and control colonies in the numbers of large workers or soldiers in them over the last 6 months (Fig. S4), and no indication anecdotally of differences before that either. However, there was an effect of the disturbance treatment on the behavior of ants in the colonies. Ants from the threat-disturbed colonies spent significantly more time in the lightened half of a Petri dish compared to ants from control colonies (*F*_1,258_ = 17.09; *P* < 0.001; Fig. 1b). In the MOR assay, ants from the threat-disturbed colonies were also significantly more threat responsive compared to those from the control colonies (*F*_1,719_ = 4.15; *P* = 0.042; Fig. 1c), and individuals that gaped their mandibles did so for significantly longer (*F*_1,182_ = 6.42; *P* = 0.012; Fig. 1d). Overall, all ants from both treatment groups, showed significantly different responses between the three stimuli in both MOR propensity and duration (*F*_2,719_ = 17.1 *P* < 0.001; Fig. S1a; *F*_2,182_ = 16.7; *P* < 0.001; Fig. S1b, respectively). Size of the focal ant showed no significant relationship with either propensity or duration of MOR response (*F*_3,719_ = 1.08, *P* = 0.36, and *F*_3,182_ = 0.99, *P* = 0.4, respectively). In the aggression assay, ants from the threat-disturbed colonies did not show a difference in propensity to be aggressive compared to ants from the control colonies (*F*_1,128_ = 0.634; *P* = 0.427; Fig. 1f), but when they did show an aggressive response, they showed a significantly higher aggression score (*F*_2,256_ = 3.39; *P* = 0.035; Fig. 1e). Colony size (volume of fungus) showed no relationships with the size of workers, the propensity or duration of MORs, phototaxis or aggression (*F*_1,257_ = 0.912, *P* = 0.340; *F*_1,962_ = 0.001, *P* = 0.983; *F*_1,321_ = 0.758, *P* = 0.385; *F*_1,258_ = 0.546, *P* = 0.461; *F*_2,253_ = 0.250, *P* = 0.779, respectively).

**Fig. 2:**
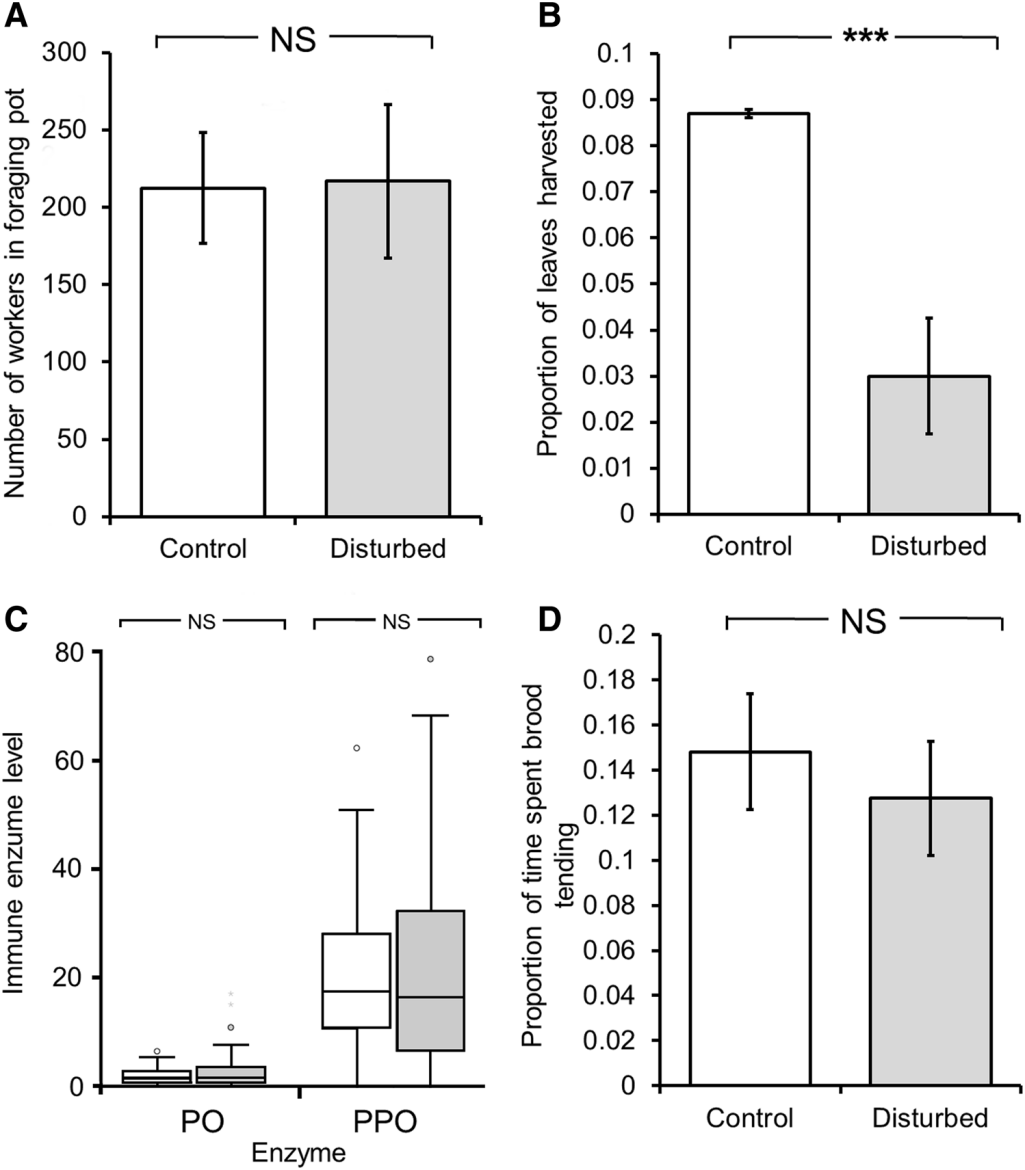
The mean ± s.e. effects of threat disturbance on: **a** number of ants present in a foraging pot after 1 h, **b** proportion of leaf material harvested after 1 h, **c** activity of the phenyloxidase (PO) and prophenyloxidase (PPO) immune enzymes, and **d** the proportion of time ants spent showing brood care behaviors over a 10 min period. Colonies either received a substantial threat-disturbance every week for 17 months or were not disturbed in this way (control colonies). *Asterisks* indicate a significant difference between threat-disturbed and control colonies (*P* < 0.001)

In the assays exploring potential effects on other traits, there was no difference between the threat-disturbed and control colonies in the number of ants in the foraging pots after 1 h (*F*_1,11_ = 0.172; *P* = 0.686; Fig. 2a), but the threat-disturbed colonies nevertheless harvested significantly less leaf material than the control colonies during the 1 h period (*F*_1,11_ = 31.8; *P* < 0.001; Fig. 2b). Colony size showed no significant relationship with either the number of ants in the foraging pot, nor (marginally) on the amount of leaf material they harvested (*F*_1,10_ = 2.16; *P* = 0.172; *F*_1,10_ = 4.67, *P* = 0.056, respectively). There was no significant effect of disturbance on the immunocompetence of ants in terms of either PO or PPO activity (*F*_2,105_ = 0.165; *P* = 0.848, and *F*_2,126_ = 0.144; *P* = 0.866; Fig. 2c), nor on the propensity of ants to pick up brood (*F*_1,248_ = 0.076; *P* = 0.784; Fig. 2d). There was no significant difference in size of colonies at the end of the experiment (*F*_1,11_ = 0.20; *P* = 0.663; mean ± s.e. size of threat disturbance and control colonies were 1087 ± 182 and 1176 ± 120 ml of fungus garden, respectively), or over the course of the experiment (Fig. S3).

### Speed of behavioral plasticity

**Fig. 3:**
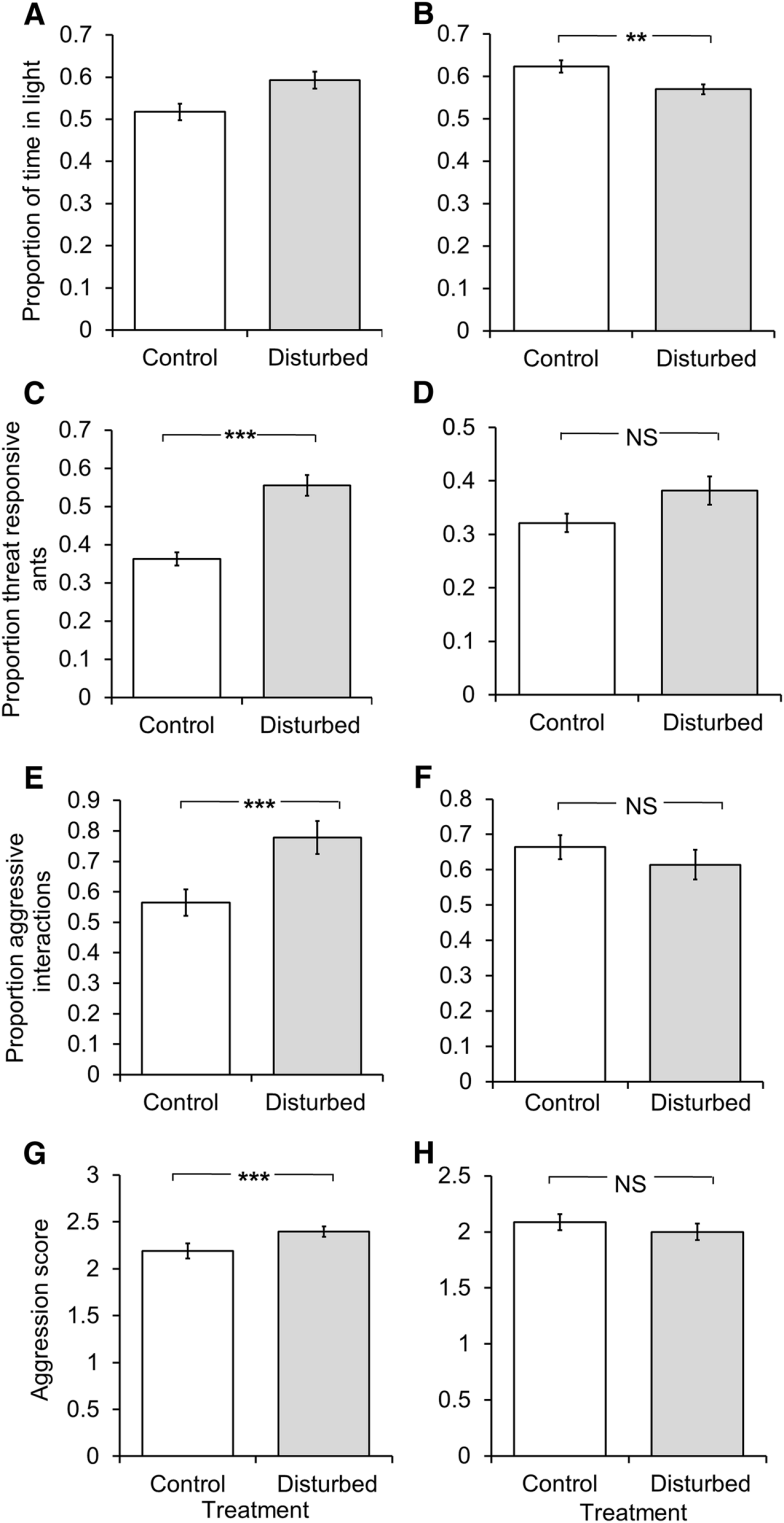
The mean ± s.e. behavioral phenotypes of *Atta* leaf-cutting ant workers from threat-disturbed colonies (*gray bars*) and control colonies (*white bars*) after short-term disturbance. *Left hand graphs* (**a**, **c**, **e**, **g**) indicate behavioral assays after 2 weeks of threat disturbance. *Right hand graphs* indicate (**b**, **d**, **f**, **h**) behavioral phenotypes 2 weeks after disturbance had ended. **a**, **b** Shows proportion of time in the light half of a half-blackened Petri dish, **c**, **d** the proportion of positive mandible opening responses (MORs) to threat stimuli (pooled responses to nestmates and non-nestmates), and **e**, **f** the average proportion of aggressive interactions and **g**, **h** the average aggression score. Colonies either received a substantial threat disturbance every day for 2 weeks or were not disturbed in this way. *Asterisks* indicate a significant difference between threat-disturbed and control colonies (*P < 0.05, **P < 0.01, ***P < 0.001) while *different letters* above *columns* in **c** and **d** indicate behavioral stimuli which differed significantly from one another in pairwise comparisons

After 2 weeks of disturbance, ants from disturbed subcolonies spent significantly more time in the light half of a Petri dish and were significantly more threat responsive than ants from control colonies (*F*_1,250_ = 6.36, *P* = 0.012, Fig. 3a and *F*_1,527_ = 20.6, *P* < 0.001, Fig. 3c, respectively). They were also significantly more aggressive than ants from control colonies, both in the propensity to show an aggressive response (*F*_1,278_ = 14.15; *P* < 0.001; Fig. 3e) and in the aggressiveness of responses (*F*_2,184_ = 3.19; *P* = 0.044; Fig. 3g). Two weeks after this short-term disturbance had ended, there was no difference between ants from disturbed and control colonies in their threat response behavior (*F*_1,530_ = 1.91; *P* = 0.179; Fig. 3d), propensity to be aggressive (*F*_1,278_ = 0.758; *P* = 0.385; Fig. 3f) or aggression score (*F*_1,176_ = 0.671; *P* = 0.414; Fig. 3h), and ants from disturbed colonies spent less, not more, time in the light half of a Petri dish (*F*_1,250_ = 7.98; *P* = 0.005; Fig. 3b).

## Discussion

Repeated threat disturbance of colonies over a prolonged period did not affect the investment by small leaf-cutting ant colonies into morphological phenotypes, but it did alter the behavioral phenotypes of individuals within disturbed colonies. Ants from disturbed colonies were significantly more threat responsive, aggressive, and phototactic than ants from control colonies, and this change in behavioral phenotype occurred after as little as 2 weeks of threat disturbance, showing how rapidly social insect colonies can behaviorally buffer themselves in the face of environmental perturbation.

The lack of any effect of frequent, and quite substantial, nest disturbances over such a prolonged period of time (17 months) on the production of soldiers or the size of large workers is at first sight surprising. Colonies of many ant species, including *Atta* leaf-cutting ants, show considerable variation within and between populations in the frequency distributions of morphological phenotypes (Davidson 1978; Oster and Wilson 1978; Wetterer 1995; Yang et al. 2004; Hölldobler and Wilson 2010; Wills et al. 2014), and it seems plausible that such intercolony variation may at least in part be a response to environmental conditions. *Pheidole* ant colonies have been shown experimentally to produce more soldiers within only seven weeks when under greater perceived threat from competitors (Passera et al. 1996). It is unlikely that the 17 month duration of disturbance in the experiment here was insufficient for a shift in caste investment given that the development time in *Atta* is about 2 months (Weber 1972), and that the disruption of morphological phenotypes can produce a change in allocation after 8 weeks in *Acromyrmex* leaf-cutting ants (Hughes and Boomsma 2007). It is also unlikely that the leaf-cutting ant colonies studied here were not genetically capable of producing larger workers or soldiers, given the relatively high intracolonial genetic diversity and genotypic variation in size propensity shown by this species (Helmkampf et al. 2008; Evison and Hughes 2011; Holman et al. 2011). The lack of an alteration to threat disturbance in the morphological phenotype distributions of colonies indicates that some other factor or constraint outweighed the stimulus from the disturbance. The exact cause is unknown, but while all colonies were healthy and old enough to produce larger workers, they were relatively small (Weber 1972). The production of larger phenotypes, particularly soldiers, will require the investment of substantially more resources than smaller workers (Oster and Wilson 1978; Segers et al. 2015). That resource limitation prevented the colonies from increasing their production of larger defensive individuals is therefore one possible explanation for the results.

Although constraints such as resource limitation may therefore limit the ability of social insect colonies to alter their morphological phenotypes, insect societies have other routes to phenotypic plasticity available to them and in this case showed an alteration of their behavioral phenotype. The results from the MOR assay indicate that all castes upregulate their individual-level threat responsiveness in response to colony threat disturbance rather than this response being limited to certain castes. Furthermore, medium-sized workers also showed an upregulation of aggression, phototaxis, and threat response behavior (Chapman et al. 2011; Bengston and Dornhaus 2014). This behavioral flexibility, particularly where aggressive or defensive behaviors are involved, could therefore offer a more adaptable and plastic alternative to a costly investment in a morphological defensive phenotype (West-Eberhard 1989; Tufto 2000; Sih et al. 2004). The short-term disturbance experiment showed that the behavioral upregulation was dynamic, being upregulated after only 2 weeks of disturbance, and downregulated again with 2 weeks of the disturbance ending. For most variables, 2 weeks after the disturbance had ended the behavior of disturbed colonies was downregulated to levels similar to those of control colonies, although phototaxis appeared to be downregulated further. This highlights the highly plastic nature of behavioral phenotypes in social insect colonies as well as the exceptional capabilities of colonies to behaviorally buffer themselves in response to environmental disturbance (Robinson 1992; Pamminger et al. 2011; Gordon et al. 2013; Yan et al. 2014).

In contrast to some other studies (Rittschof and Robinson 2013), the behavioral upregulation showed no evidence of habituation, with the level of upregulation after 17 months being very similar to that after 2 weeks. Anecdotally there was no evidence of morphological habituation either, with the number of large workers remaining small in both treatment groups over the course of the experiment (Fig. S4). The lack of habituation may perhaps be due to the severity of the disturbance in the experiment, and of the predator threat that exposure of the fungal garden would indicate in nature (Rao 2000). Interestingly, there was some evidence from our limited assays that the change in behavioral phenotype may affect other traits, with workers from threat-disturbed colonies harvesting less material than those from control colonies during the brief 1 h foraging assay, in spite of the same number of ants having been recruited to the food. There was no difference in the size of fungal gardens of threat-disturbed and control colonies over the course of the experiment, showing that any effect on foraging rate did not have an effect on colony growth in the competitor-free, food-rich environment of the experiment. It would therefore be interesting to see whether threat-disturbed colonies have lower foraging rate over a longer time period and whether this would impact colony growth, either when competitors are present or when food is more transiently available.

This study highlights that changes in behavioral phenotype may offer a more rapid and flexible alternative to changes in morphological phenotypes. Social organisms are particularly interesting in this regard because phenotypes can be expressed at both individual and group levels (Korb and Heinze 2004; Chapman et al. 2011; Dornhaus et al. 2012), and further investigation of the dynamic relationships between phenotypes and environmental cues is likely to be very useful in elucidating the evolution and dynamics of phenotypic plasticity.

## Electronic supplementary material

Below is the link to the electronic supplementary material.

Supplementary material 1 (XLSX 185 kb)
